# Influence of oral health on mucositis in patients undergoing hematopoietic progenitor cell transplantation (HPCT)

**DOI:** 10.4317/medoral.16997

**Published:** 2011-12-06

**Authors:** Ana Hernández-Fernández, Ricardo E. Oñate-Sánchez, María C. Cabrerizo-Merino, Felipe de Arriba de la Fuente, Inmaculada Heras Fernando, Vicente Vicente García

**Affiliations:** 1 Associate Lecturer. Faculty of Dentistry, University of Murcia; 2 Senior Lecturer. Faculty of Dentistry, University of Murcia; 3 Associate Lecturer. Health Sciences Clinic, Faculty of Dentistry, University of Murcia; 4 Hematology and Oncology Services, Morales Meseguer Hospital

## Abstract

Aims: To establish whether or not the state of patient oral health can influence the occurrence and/or severity of oral mucositis during hematopoietic progenitor cell transplantation (HPCT). Materials and Methods: The study included 72 patients awaiting HPCT. Prior to transplantation, clinical exploration and radiology were carried out and oral photographs were taken. This evaluated the extent of caries present, the number of missing teeth and the number of dental fillings in each patient; CAO (Caries and Obturations Index) DMFS (Decayed, Missing, and Filled Surfaces) and Restoration Indices were calculated. Gingival pathology was also examined by means of the Ainamo and Bay Gingival Bleeding Index. O’Leary’s Plaque Index was used to evaluate the level of patient oral hygiene. This data was analyzed to see if it exercised any influence on the mucositis grade suffered during HPCT. Results: 96,87% of patients suffered some degree of mucositis during their treatment by the Transplant Unit. The grade of mucositis was seen to be influenced by the number of missing teeth (ANOVA p<0.016) and by the DMFS Index (ANOVA p< 0.038). Although this was not one of the aims of this study, patient age and the administration of colony-stimulating factors were also seen to influence these clinical manifestations. Conclusions: The state of prior oral health can influence decisively the mucositis suffered during transplantation.

** Key words:** Hematopoietic progenitor cell transplantation, mucositis, state of oral health.

## Introduction

An ageing population, the increased occurrence of certain malignant tumors and the longer survival rates of cancer sufferers, has lead to a significant increase in the numbers of patients suffering malignant neoplasias (1).

Amongst these neoplasias, hematologic neoplasias are prevalent, leukemia and lymphoma being the most frequent. Treatment of hematologic aplasia includes chemotherapy (CT), radiotherapy (RT) and often a combination of both. Most of these patients will also undergo hematopoietic progenitor cell transplantation (HPCT) not only for the eradication of the cancer but also to soften the side effects of the high levels of radiation and the cytostatics involved in treatment. 

During the transplant period and immediately afterwards, patients may present a variety of symptoms, of which oral mucositis is one of the most frequent and debilitating. 

Within the field of oncology, mucositis is understood as the inflammation of the oral mucosa characterized by redness and pain, a common side effect of oncotherapies. 

The occurrence of mucositis has always been explained as a combination of factors associated with and giving rise to this situation. These factors include: high index of epithelial buccal cell replacement, a direct effect of radiation and cytostatics on the tissues involved (toxicity), vascular disturbances that compromise healing processes, altered immune responses due to myelosuppression and lastly, modifications to buccal flora due to hypoplasia and the administration of prophylactic drugs (antibiotics, antifungals, and antivirals). 

Several studies (2-6) suggest that various factors that may influence the grade of mucositis in patients undergoing HPCT, although some of these have not been confirmed: 

- Intensity of the conditioning regime prior to HPTC. 

**Table 1:**
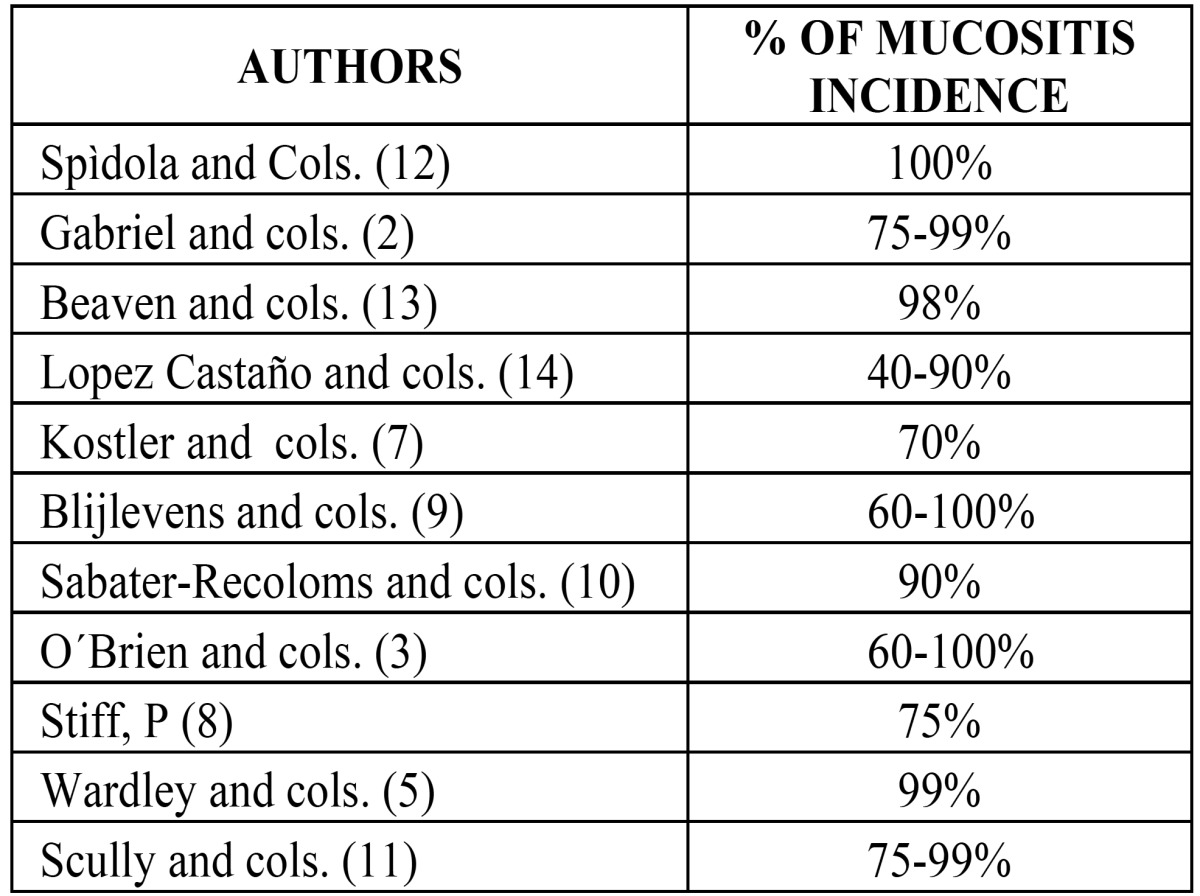
Frequency of mucositis. Incidence according to different authors.

- Transplant type (allogeneic or autologous). 

- The origin of the cells implanted (when this is bone marrow mucositis is more severe than when the source is blood). 

- Use of colony-stimulating factors, also known as myeloid growth factors (the grade of mucositis appears to diminish, although researchers have not reached agreement on this). 

- Patient age (in young patients mitotic rates are higher, but an increase in mucositis severity in elderly patients has also been observed). 

- Use of total body radiation (TBR). 

Mucositis may be caused both by radiotherapy and chemotherapy and a combination of the two usually increases the severity of the symptoms. (7,8)

Clinical manifestations may vary, ranging from erythema or localized ulcers to the total loss of the epithelium with secondary hemorrhages and intense pain, which may give rise to (2, 9-11):

- Nutritional disturbance.

- Speaking difficulties.

- Difficulties for drug administration by mouth.

Clearly this represents a major reduction to the patient’s quality of life as well as increased duration of fever, parenteral nutrition, opiate administration and risk of infection, with all the related repercussions and higher hospitalization costs (3-5, 10). 

According to some researchers (12), oral and intestinal mucositis occurs in 100% of patients subjected to HPTC following the conditioning regime. Others (2,3,5, 7- 10, 13,14) do not put the percentage at 100% but do state that it has a very high rate of incidence ( Table 1). Furthermore, mucositis occurs more frequently in cases of hematologic tumors than solid tumors (14). This is due to the fact that the intensity and duration of myelosuppression is two or three times greater in patients with hemopathies and those subjected to hematopoietic progenitor cell transplantation (15).

The World Health Organization established a scale of mucositis severity based on a number of parameters, which has been in use since 1979 (16). 

The WHO (17) states that buccodental health continues to be one of the major problems for public health the world over. For this reason, in 2003 the WDF, the WHO and the IADR, drew up a plan fixing a series of dental health targets to be met in the new millennium (by 2020) (17). Amongst these are: reduction in the death rate due to oral and craniofacial disease, reduction of the impact of oral pathologies on systemic disease and the integration of buccodental health care into other medical disciplines. 

In accord with these last two objectives, the present study focuses on evaluating the level of oral health in patients prior to HPCT and its influence on mucosa affectation during the transplantation procedure. 

 Objectives

The objective of this study was to discover whether the state of oral health (evaluated in terms of caries, gingival disorders and plaque) influences the severity of oral mucositis during hematopoietic progenitor cell transplantation.

## Material and Methods

 The study included 72 patients who were due to receivehematopoietic progenitor cell transplantation. They were referred to the Special Patients Teaching Unit at the University Dental Clinic by Morales Meseguer Hospital Oncohematology Service. 

Oral exploration was performed at the University Dental Clinic in morning sessions at least fifteen days before patients were to be hospitalized at the Transplant Unit. 

Study inclusion criteria were: 

- Patients who had been diagnosed with either cancers of different types and at different locations or myelodysplastic syndrome.

- Treatment of these pathologies was to include hematopoietic progenitor cell transplantation. 

- Patients with teeth so that the state of oral health could be assessed; completely edentulous patients were excluded.

Having obtained informed consent from each patient, clinical and radiological explorations were performed and oral photos were taken. 

The materials used for clinical exploration were: patient clinical history compiled by the Teaching Unit, an exploration kit, plaque disclosing tablets and other related disposable materials. Patient clinical histories were designed by the Special Patients Units to meet the requirements of the present study. The exploration kit consisted of: two dental mirrors with stainless steel handles (Roeko®) and Nº 5 mirror (Actual®), dental tweezers 2885 (Martin®), a 17-22 (Hu-Friedy®) exploratory probe and a PCP 11 5B (Hu-Friedy®) periodontal exploratory probe. The plaque disclosure tablets used were Plac Control® (Dentaid).

Radiographic exploration consisted of orthopantomograms (OPG), which were taken before oral exploration began. The orthopantomography equipment used was the PM 2002 CC (Plamenca®).

Radiographic images necessarily included all the details to be analyzed for the purposes of the study with sufficient image quality for clear visualization of these features and structures. 

The photo camera used was a Yashica Reflex model FX-3 fitted with a 100 mm Yashica® macro lens and three extension tubes, to which an annular flash was attached. Intraoral mirrors of various sizes and shapes were used for taking the photos, as well as lip separators for children and adults. 

Data collection was performed by a single practitioner and was recorded by a second member of the team who acted as auxiliary. Both the examiner and the data compiler were fully trained and all evaluations were calibrated according to WHO criteria by means of the Kappa coefficient of determination. 

The patient was received at the dental clinic and personal details were recorded before taking the patient for orthopantomography (OPG). An odontogram was filled out which included gingival data. Oral photos were then taken. Lastly, each patient was given a disclosure tablet for evaluating the level of oral hygiene and when the session had come to an end the indices were calculated. 

Diagnosis of caries followed WHO criteria. Any case in which it was not clear whether or not these criteria had been met was regarded as caries-free. As well as registering the extent of caries, number of missing teeth and number of fillings per patient, the following indices were calculated: CAO (Caries and Obturations Index) DMFS (Decayed, Missing, and Filled Surfaces) and Restoration Indices. For gingival examination, gingival pathology was examined by means of the Ainamo and Bay Gingival Bleeding Index (simplified by Lindhe). To generate this index the following classifications were established: 

- Bleeding Intervals 0%

Gingival Inflammation absent

- Bleeding Intervals > 0 – 10%

Gingival Inflammation slight

- Bleeding Intervals > 10 – 50%

Gingival Inflammation moderate

- Bleeding Intervals > 50%

Gingival Inflammation severe

In addition to the Bleeding Index, an O’Leary plaque index was evaluated. This index was always calculated after photographs had been taken. This is also known as the Oral Hygiene Index and has the following classifications: 0-10% excellent; 10-25% good; 25-75% regular; >75% poor.

Further data was obtained from the Oncology Clinic medical notes taken during the transplantation procedure which included: presence or not of mucositis, grade of mucositis according to the WHO mucositis grading scale, source of hematopoietic progenitor cells and the administration or not of colony growth factor. The WHO grading scale is as follows: Grade 0: None; Grade 1: Soreness and/or erythema, no ulceration; Grade 2: Erythema, ulcers. Patients can swallow solid diet; Grade 3: Ulcers, extensive erythema. Patients cannot swallow solid diet; Grade 4: Oral mucositis to the extent that alimentation is not possible.

All the data compiled was subjected to statistical analysis using SPSS software. Variables were analyzed both descriptively and analytically. For the descriptive study, qualitative variables were calculated with frequency distribution, whilst for quantitative variables, means were calculated as a centralization measure; standard error of the mean and standard deviation were used as measures of dispersion; and maximums and minimums were used to establish ranges. Relations between qualitative and quantitative variables were performed using the student t-test whenever two averages were involved; when more than two were involved the analysis of variance test (Anova) was applied. For relationships between qualitative variables, contingency tables were used and Pearson’s chi-squared test was applied; analysis of residues was also performed where necessary. In all cases, a difference between groups or a relation between variables was taken as statistically significant when the significance level was less than 0.05 (p<0.05).

**Figure 1:**
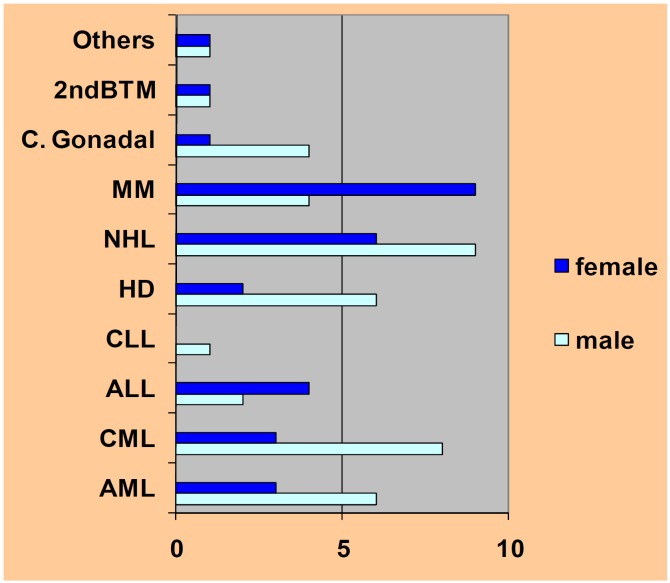
Pathology distribution by gender. 2nd BMT= 2nd bone marrow transplant, C= carcinoma, MM= Multiple Myeloma, NHL= Non Hodgkin Lymphoma, HD= Hodgkin Disease, CLL= Chronic Lymphocytic Leukemia, ALL= Acute Lymphocytic Leukemia, CML= Chronic Myeloid Leukemia, AML= Acute Myeloid Leukemia.

**Figure 2:**
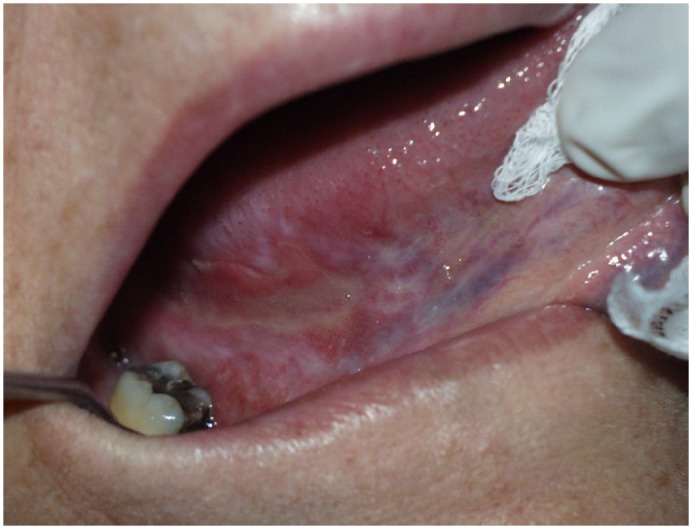
Mucositis on tongue dorsum during HPCT.

**Figure 3:**
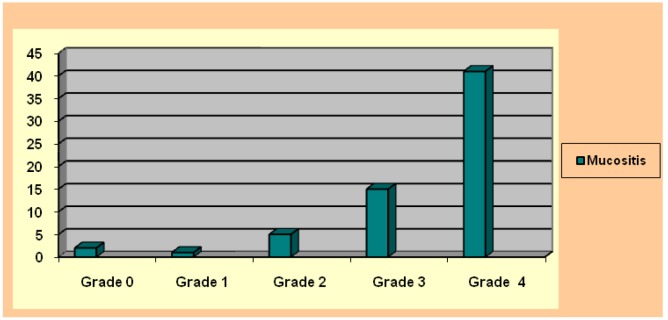
Mucositis grades according WHO classification.

**Table 2:**
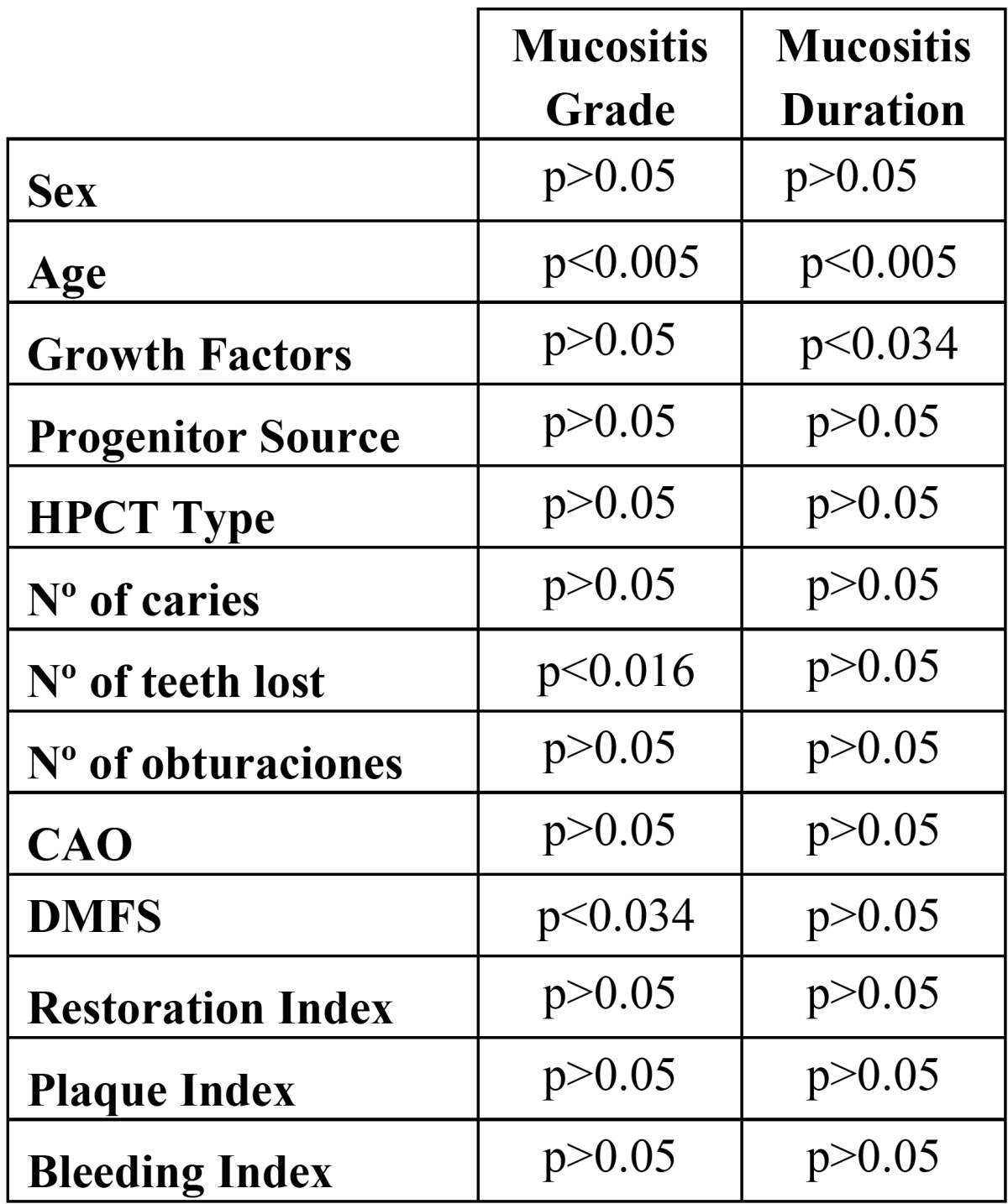
Factors relating to oral mucositis during HPCT.

## Results

 Of the 72 subjects, 42 were men and 30 were women. The average age of subjects was 42.2 ± 1.9 years, the youngest patient being 12 years old and the oldest 69. 

All subjects were due to receive HPC transplants and had been diagnosed with different neoplasias although the majority had blood dyscrasias. 37.5% of subjects had leukemia, 31.9% had been diagnosed with some type of lymphoma and 18% suffered multiple myeloma (Fig. 1). 

All were to undergo either autologous transplantation (42 subjects) or an allogeneic transplant (30 subjects). The majority (93.54%) were given transplants using progenitor cells from blood collected by means of aphaeresis. Only four were to be transplanted with bone marrow stem cells.

Only two patients did not suffer oral mucositis during the procedure. This meant that 96.87% did suffer some grade of mucositis during the period of hospitalization at the Transplant Unit (Fig. 2). The most frequent mucositis value was grade 4 found in 64.06% of patients. In descending order, the second most frequent value was grade 3 23,43% (Fig. 3). With regard to mucositis duration, this variable was not recorded in all the case notes supplied by the Transplant Unit. However, this information was made available in 52 cases, of which most fell within the 8 – 14 or 15 – 21 day classifications, so that the rest were in the <7 day class. 

65% of subjects were administered colony-stimulating factors whilst 35% were not. 

Initial oral health was found to be generally deficient: 83% of patients showed caries, 67% of subjects had some tooth/teeth missing and 40 subjects had received no dental restoration treatment whatsoever (56%). The mean average CAO Index was 10.37 ± 0.77. Gingival bleeding was scarce; only 20.3% of patients showed a bleeding index greater than 50%, a value signifying a high level of gingival inflammation. 78.12% of the sample group showed very poor levels of oral hygiene with plaque accumulation on 75% of tooth surfaces. 

Evaluating the interdependence between mucositis and the other parameters analyzed ( Table 2) it was found that: 

a) *Mucositis/oral health relation:* The grade of mucositis suffered was seen to be influenced by the number of missing teeth (ANOVA p<0.016) and by the DMFS Index (ANOVA p< 0.038) with patients presenting grade 2 mucositis having more missing teeth and higher DMFS indices than patients with grade 3 mucositis, who in turn showed higher indices than patients suffering grade 4 mucositis. 

b) *Mucositis/periodontal health relation:* The bleeding index was found to be unrelated to either the grade or duration of mucositis suffered during transplantation procedures.

c) *Mucositis/oral hygiene relation:* Oral hygiene was not found to influence mucositis.

d) *Mucositis/other factors relation:* The study also considered the possible dependence between mucositis and other factors that might exercise some influence in the appearance of symptoms, even though this was not a main study objective. Such factors included: treatment received prior to the transplantation procedure, the transplant itself, the source of hematopoietic progenitor cells, the administration of myeloid growth factors and patient age. Transplant type was not found to influence the grade of severity or duration of mucositis suffered. Relating patient age with mucositis grade and its duration, a statistically significant relation was found (ANOVA p<0.005). The patient group with the youngest average age showed higher mucositis grades and longer duration. The progenitor cell source factor was not seen to have any influence on the grade or duration of mucositis. Nor did the administration of colony-stimulating factors during transplantation procedure. However, a significant relation (Chi-squared p<0.034) was found with regard to the duration of mucositis symptoms, so that the administration of these growth factors is associated with a reduction in the duration of mucositis. 

## Discussion

 Cancer is one of the major health problems the world over. According to the World Health Organization (WHO), around eleven million people are diagnosed with some kind of malignant tumor every year and 6.7 million lose their lives. Of these 6.7 million, 56% are men and 44% are women. At present, it is estimated that there are 22 million people who receive medical attention for neoplasia. 

In Spain, one in three men and one in five women will develop some kind of cancer before the age of 74, indicating that it is a very common disease. For this reason it is important to study and analyze all possible problems that cancer patients may suffer in all aspect of their lives. Knowing what these problems are is the first step towards solving them. 

As HPCT produces good results, it is currently used for treating diverse types of hematologic diseases, not only blood neoplasias but also certain solid tumors. For this reason the number of patients receiving this type of therapy has risen during recent years. Given that mucositis is one of the most frequent side-effects of this treatment, and the one that causes the greatest discomfort, it is important reach an in-depth understanding of this pathology in order to administer the best possible palliative care during HPC transplantation. 

In terms of gender, age and tumoral pathology, the study sample was representative of patient groups in the literature reviewed prior to designing this study, a fact that facilitated comparison of our findings with those of other research. Concordance with the articles reviewed was found in terms of: 

- Sex and age of the study subjects 

- Tumoral pathology 

- The transplant type to which subjects were subjected 

- Hematopoietic progenitor cell source. 

With regard to study subjects’ oral health, a high number of teeth with caries was found, higher than would be expected amongst the population at large according to the last survey of buccodental health carried out in Spain in 2005; this was accompanied by a low rate of dental restoration. In the same way, gingival health was also deficient and the level of oral hygiene very poor. Furthermore, the state of oral health was found to worsen as patient age increased. Given the presence of so many possible septic foci in these patients, who are highly susceptible to infection, it is important to recognize the need for and importance of providing prior dental care for all patients who are to be treated with HPCT. 

Regarding the analysis of mucositis grades, it is not easy to make comparisons across the range of research published due to the wide variety of scales and systems for quantifying mucositis symptoms in use in clinical practice (14,18). Nevertheless, it would seem that increasingly many researchers are adopting the scale established by the WHO, which is now in general use (4).

According to Wardley et al. (5), the mucositis occurring in HPCT patients is usually grade 3 or 4 (64.06% and 23.43% respectively).

Similarly, in a literature review dealing with oral mucositis, Gabriel et al. (2) refer to a number of authors who affirm that the majority of patients suffer severe levels of mucositis during HPCT. In contrast to this review, a study carried out by Sabater-Recoloms et al. (10) found that the grade of mucositis most frequently suffered was grade 1 on the WHO scale (44,3%), and the more severe grades appeared in lower proportions. This might be due to the fact that in their study the patients were selected without distinguishing between patients who were to receive chemotherapy and those who were to undergo HPCT. In the present study, all patients underwent HPCT and so the conditioning regime was more intense and mucositis severity greater. 

Although there are numerous studies of the incidence and intensity of mucositis during HPCT, we have been unable to find much research into its duration. Filicko et al. (4) state that it usually occurs between the second and eighteenth day of the transplantation procedure, whilst Gabriel et al. (2) place it between the third and tenth days. In this study, it was found that most of the subjects suffered mucositis between the eighth and twenty-first days, a finding that differs from other studies. 

In the present study, the grade of mucositis was inversely related to the number of missing teeth and DMFS Index, in other words, the greater the number of teeth lost and the higher the DMFS Index, the lower the mucositis grade. In the case of the number of missing teeth, this might be due to the fact that tooth friction traumatizes the inflamed mucosa, given that the softer or more liquefied diet associated with edentulism would eliminate food as a traumatizing factor. 

Statistical differences were not found between plaque accumulation and the grade or duration of mucositis suffered. This contradicts the findings of other authors (5,10,14,15). However, there are some studies that state that subjecting patients to rigorous regimes of oral hygiene does not produce very substantial benefits with regard to mucositis (19). Even though many articles published regard oral hygiene as a conditioner for the development of higher or lower grades of mucositis, few studies have been carried out that examine the veracity of this. Given that all the patients who took part presented high plaque levels, we wonder if perhaps they paid little attention to oral hygiene due to their greater concern in the face of the much more serious systemic pathology that is cancer. 

A number of authors (4,6,19) affirm that the grade and duration of mucositis are greater in patients who receive allogeneic bone marrow transplantation (BMT) compared to those who receive autologous BMT. The findings of the present study are unable to confirm this, although it can be said that this is likely, given that alloBMT patients are subjected to a more aggressive conditioning regime. In contrast to other studies reviewed (3,5,6), the source of hematopoietic progenitor cells was not found to influence mucositis.

The use of myeloid growth factors during transplantation did not modify mucositis grade but was seen to reduce the number of days of its duration. This coincides with the fact that growth factors are administered chiefly to reduce the days of neutropenia that the patient suffers following transplantation (8) and several authors (7,8) have inversely related neutrophil figures with the incidence of oral mucositis. 

However, authors have been unable to reach agreement as to whether the use of granulocyte colony-stimulating factor and granulocyte macrophage colony-stimulating factor benefit patients with regard to mucositis. In this way, whilst many authors (4, 6- 8, 11) agree on the reduction not only of duration but also the intensity of mucositis affectation, others (5) maintain that it is counterproductive given that its use requires more aggressive conditioning treatments, which can be life threatening. Further research is needed to find out whether myeloid growth factors influence the incidence, duration and severity of mucositis, given that some of the articles reviewed fail to indicate the ways in which its influence is exercised in terms of these variables. 

Mucositis severity also has an inverse relation to age (the younger the patient, the higher the mucositis grade). It may be that younger tissues are more affected by oncological treatment, but nevertheless, as people tend to lose teeth with advancing age, this will also have an effect on mucositis severity as described earlier. 

With regard to mucositis duration and patient age, it was clear that the younger the average age, mucositis duration was longer. This could be due to the fact that, with the greater replacement of epithelial cells in young subjects, this may make them a more hospitable target for cytostatics. Many authors (7,8,10,12) include age as a risk factor for oral mucositis, but none of them have made the nature of this relation clear.

## Conclusions

 In the group of patients who took part in this study, oral health levels were found to be poor in terms of plaque-related diseases, with high levels of caries and teeth lost. Age exercises a decisive influence on the parameters determining caries, so that the older the subject the worse the state of oral health. The Restoration Index was also found to be lower the older the patient. 

Both dental and gingival health were worse in this study group than amongst the Spanish population at large according to the last survey of buccodental health carried out in Spain. 

Oral mucositis duration was seen to diminish with administration of colony-stimulating growth factors. Both the duration and grade of mucositis increased as patient age got younger. 

The higher the number of missing teeth and the higher the DMFS Index, the lower the grade of oral mucositis suffered during HPCT. 

Oncohematological Services should work more closely with dental services in order to reduce, as far as possible, mucosa complications during HPCT, so that patients enter the Transplant Unit in a better state of oral health.
